# A functional SNP rs12718466 in APOA1 promoter modulates gene expression *via* interaction with SOX7

**DOI:** 10.1016/j.jbc.2026.113101

**Published:** 2026-04-27

**Authors:** Yuichi Aita, Yoshinori Takeuchi, Yukari Masuda, Zahra Mehrazad Saber, Samia Karkoutly, Duhan Tao, Chen Ye, Tsolmon Mendsaikhan, Rika Saikawa, Yasuyuki Kondo, Yuki Murayama, Akito Shikama, Takashi Matsuzaka, Hitoshi Shimano, Yasushi Kawakami, Naoya Yahagi

**Affiliations:** 1 Division of Endocrinology and Metabolism, Department of Medicine, Jichi Medical University, Tochigi, Japan; 2 Nutrigenomics Research Group, Institute of Medicine, University of Tsukuba, Ibaraki, Japan; 3 Department of Internal Medicine (Endocrinology and Metabolism), Institute of Medicine, University of Tsukuba, Ibaraki, Japan

**Keywords:** functional SNP, high-density lipoprotein cholesterol, transcription factor

## Abstract

Plasma concentration of high-density lipoprotein cholesterol (HDL-C) is among the most important risk factors for coronary artery disease and apolipoprotein A1 (APOA1) is an essential apolipoprotein that constitutes HDL. However, few comprehensive searches have been conducted to identify noncoding functional SNPs around the APOA1 gene. In this study, we report the identification of a functional SNP, rs12718466, which influences hepatocyte-specific APOA1 gene expression. Furthermore, we identified SRY-box transcription factor 7 (SOX7) as the transcription factor interacting with the rs12718466 SNP, using a novel screening method Transcription Factor Expression Library scan, which employs a comprehensive library of mouse transcription factors. SOX7 binding is allele-dependent, with stronger binding to the normal allele leading to increased APOA1 transcription. *In vitro* experiments in hepatocytes and *in vivo* experiments in mice confirmed that overexpressing SOX7 increased APOA1 expression, while knocking it down decreased both APOA1 gene expression and plasma HDL-C levels. Our research demonstrates that rs12718466 is a functional SNP that modulates APOA1 gene expression through its interaction with SOX7, thereby affecting plasma HDL-C concentrations.

Plasma concentrations of total cholesterol, low-density lipoprotein cholesterol, high-density lipoprotein cholesterol (HDL-C) and triglycerides (TGs) are among the most important risk factors for coronary artery disease and are targets for therapeutic intervention (1). A previous meta-analysis of 46 lipid genome-wide association studies (GWAS) involving over 100,000 individuals of European descent identified 95 loci that showed a genome-wide significant association with at least one of the four tested traits, total cholesterol (TC), low-density lipoprotein cholesterol, HDL-C, and TGs, demonstrating that many of the loci will yield insights into the biological underpinnings of lipid metabolism (2). Of the 95 loci identified, rs964184, the lead SNP located within the APOA1-C3-A4-A5 gene cluster, was found to have the largest effect on TG levels as well as the fourth largest effect on HDL levels after the lead SNPs in CETP, LPL, and HNF4A.

APOA1 is an essential apolipoprotein that constitutes HDL (3). Consistently, GWAS focusing on HDL levels have identified several SNPs mapped to the coding region of the APOA1 gene. For example, missense mutations in the APOA1 gene, such as rs12718465, rs199759119, and rs138407155 were associated with reduced HDL levels (4, 5, 6). However, there have been few comprehensive searches to identify noncoding functional SNPs around the APOA1 gene. According to a systematic review aimed at clarifying the landscape of GWAS validation, only 6.6% of total published GWAS articles successfully validated noncoding functional variants (7), demonstrating the difficulty of identifying functional SNPs from lead SNPs in general.

To greatly facilitate the functional analysis of SNPs, we have developed a new method named Transcription Factor Expression Library (TFEL) scan (8); we constructed a comprehensive expression library of transcription factors in the mouse genome, the TFEL, and developed a method of identifying upstream transcription factors through expression cloning using the TFEL (TFEL scan method). Using the TFEL scan method, we demonstrated that SNP rs7074440 is the functional SNP altering TCF7L2 gene expression by interacting with the transcription factor C-FOS in hepatocytes (9).

Therefore, we aimed to identify the functional SNPs mapping to the transcriptional regulatory region of APOA1 gene as well as the transcription factors acting there using the TFEL scan method.

## Results

### Selection of potential functional SNPs in APOA1 promoter region

**Figure 1:**
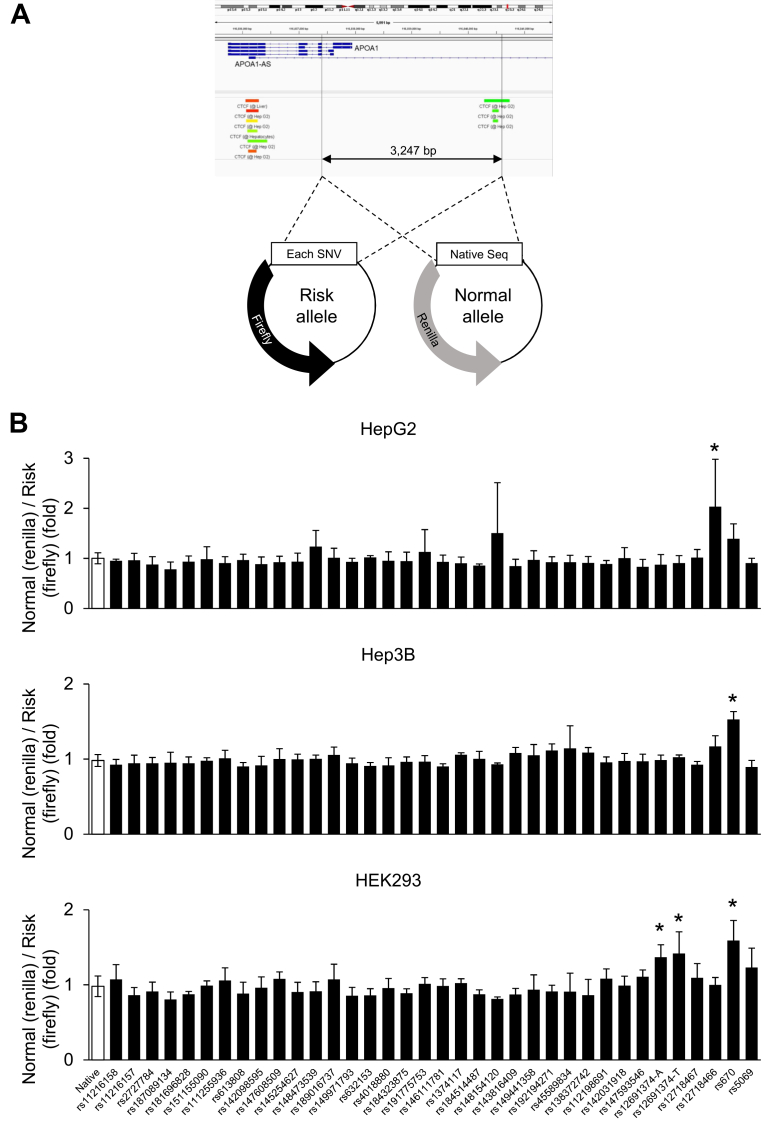
**Functional screen of SNPs around the APOA1 promoter.***A* and *B*, thirty-five SNPs of the APOA1 promoter, each harboring only one SNP (risk allele), were inserted into the firefly luciferase reporter plasmid. The APOA1 promoter without any SNPs (normal allele) was inserted into the renilla luciferase reporter plasmid (*A*). These plasmids were cotransfected into cultured cells (HepG2, Hep3B, and HEK293), and luciferase activity was subsequently measured (*B*) (n = 3–5). Data are expressed as means ± SD. Significant differences were assessed using ANOVA, when indicated by appropriate *p* values (*p* < 0.05), by Dunnett *post hoc* test. The differences were considered to be significant if *p* < 0.05. For rs12691374, two risk alleles (A and T) were generated and assayed independently; results are presented as indicated. APOA1, apolipoprotein A1.

Apolipoprotein genes are clustered on human chromosome 11. As shown in Figure S1, APOA1, APOC3, APOA4, and APOA5 are aligned, surrounded by SIK3 and ZPR1. Liver Hi-C data reveal that these genes form one topologically associating domain (10). When this domain is observed microscopically, it can be divided into several sections. The APOC3 enhancer and APOC3/A4/A5 promoters reside in the same chromatin loop, where the APOC3/A4 promoters are pointed toward the enhancer, whereas the APOA1 promoter is present in the different loop (11). Because APOA1 gene expression may be regulated independently of other apolipoprotein genes, we decided to search for potential functional SNPs in the approximately 3.3 kb region between the upstream CCCTC-binding factor (CTCF) binding site and the transcription start site (TSS) shown in Figure 1*A*. To identify potential functional SNPs in this region, we selected 14 common SNPs (minor allele frequency (MAF) > 0.01) as well as 21 rare SNPs (MAF ≤ 0.01) that had at least one linked SNP with r^2^ > 0.8 in African, American, Asian or European populations, based on HaploReg information (12). For clarity, “linked SNP with r^2^ > 0.8” indicates strong linkage disequilibrium within the neighboring genomic region, as annotated in HaploReg. Under such linkage disequilibrium structure, low-frequency variants can nevertheless tag putative functional regulatory elements. As a result, a total of 35 SNPs were included in the screening process (Table S1).

### Functional screen of SNPs in APOA1 promoter region shows a hot spot at rs12718466 in hepatocytes

The APOA1 pro-Firefly Luc and a plasmid carrying one SNP by mutagenesis were transfected into cultured cells (HepG2 and Hep3B hepatocytes and HEK293 nonhepatic cells) together with the APOA1 pro-Renilla Luc. As a result, a significant difference due to a single nucleotide difference was observed in the ratio of luciferase activity in each of the three cultured cells (Fig. 1*B*). In HepG2 hepatocytes, rs12718466 significantly increased the ratio of luciferase activity, indicating that the normal allele had stronger transcriptional activity than the minor allele. On the other hand, rs670 significantly increased the ratio in Hep3B hepatocytes, and this increase was also induced in HEK293 nonhepatic cells. In HEK293 cells, rs12691374—which carries two nonreference (risk) alleles, A and T—significantly increased the ratio; both A and T variants were tested separately in our mutagenesis/reporter assays and showed a similar direction of effect. Consistent with these screening results, endogenous APOA1 is undetectable in HEK293, weak in Hep3B, and robust in HepG2 (Fig. S2), providing physiological context for the HepG2-specific detection of rs12718466. To further assess hepatocyte specificity across species, we performed an analogous reporter-based screen in AML12, a mouse hepatocyte cell line; rs12718466 again showed the most significant allele-dependent response among all SNPs tested (Fig. S3). These results identified rs12718466 as the SNP in the APOA1 promoter region that may contribute to hepatocyte-specific APOA1 gene expression.

### TFEL genome-wide screen of trans-acting factors for rs12718466 identifies SOX7

**Figure 2:**
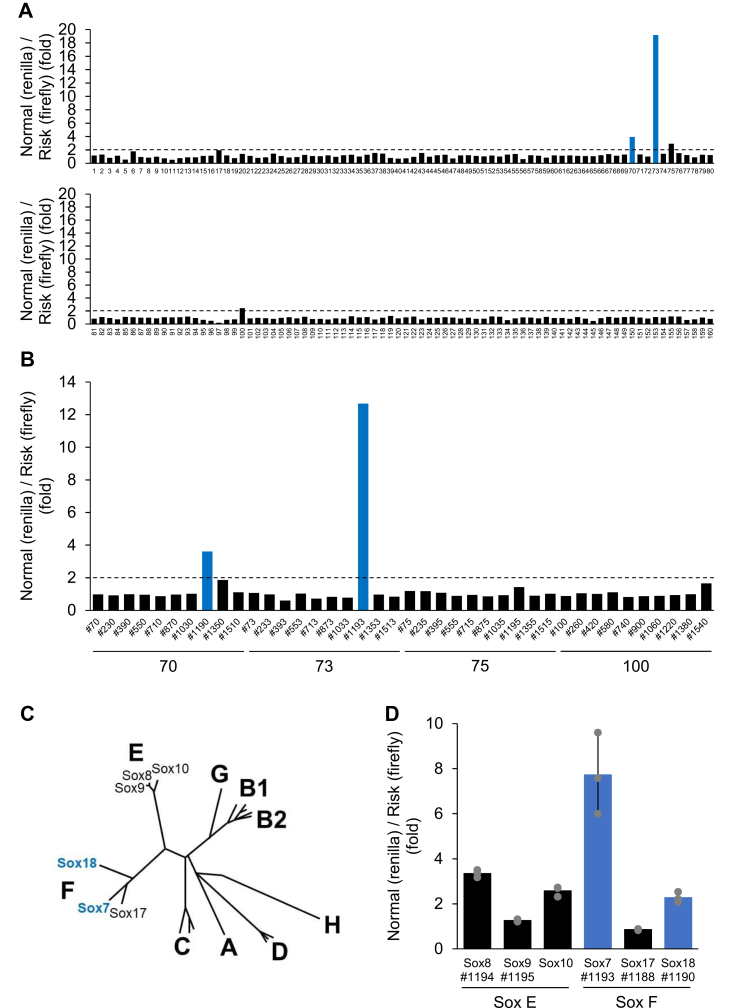
**TFEL genome-wide screen of trans-acting factors for rs12718466 identifies Sox7.***A* and *B*, the screen for transcription factors that bind to rs12718466 was performed using the Transcription Factor Expression Library (TFEL). Of 1588 transcription factors, 10 clones were transfected simultaneously in the primary screen (*A*), and only one clone was transfected in a secondary screen (*B*). HEK293 cells were cotransfected with the TFEL clones and reporter plasmids (APOA1 pro-Firefly Luc-rs12718466x3 containing the risk allele and APOA1 pro-Renilla Luc-rs12718466x3 containing the normal allele). Cells were lysed 24 h after transfection, and then firefly and renilla luciferase activities were measured. These discovery-phase screens were performed as single-run assays (n = 1) to efficiently triage candidates from the TFEL library. *C*, phylogenetic tree adapted from the Sox HMG-domain phylogeny by Kamachi and Kondoh (13). No *de novo* computational reconstruction was performed for the present figure. *D*, the indicated expression plasmids of Sox proteins were cotransfected with reporter plasmids (APOA1 pro-Firefly Luc-rs12718466x3 containing the risk allele and APOA1 pro-Renilla Luc-rs12718466x3 containing the normal allele) in HEK293 cells. Cells were lysed 24 h after transfection, and then firefly and renilla luciferase activities were measured (n = 3). APOA1, apolipoprotein A1; SOX7, SRY-box transcription factor 7.

To screen for transcription factors that bind to rs12718466, APOA1 pro-Firefly Luc-rs12718466x3 and APOA1 pro-Renilla Luc-rs12718466x3 were cotransfected together with the TFEL clones, and then luciferase activity was measured. As shown in Figure 2*A*, in the primary screen in which 10 clones were transfected simultaneously, four groups exceeded the threshold of two-fold increase. In this screen, each 10-clone pool was assembled at random, rather than by structural class or function of transcription factor, to minimize pooling bias. In a secondary screen, when clones from these four groups were transfected individually, the transcription factors #1193 and #1190 increased the ratio of luciferase activity, resulting in clearly exceeding the threshold (Fig. 2*B*). However, as is sometimes observed, no transcription factors in the other groups increased the ratio when clones were transfected individually.

As a result of TFEL genome-wide screen, we identified two transcription factors: Sox7 (#1193) and Sox18 (#1190). Sox proteins are classified into groups A-H, depending on the amino acid sequence of the HMG domain (13). As shown in Figure 2*C*, both SRY-box transcription factor 7 (Sox7) and Sox18 belong to group F along with Sox17. Group F are closely related to group E (Sox8, Sox9, and Sox10). Therefore, we reevaluated the ratio of luciferase activity ratio for transcription factors in these groups. This led to the identification of Sox7 as a transcription factor that activates the cis-element surrounding rs12718466 (Fig. 2*D*). According to public databases such as RefEx (14), all three transcription factors in group F are expressed in human liver, whereas Sox8 and Sox10 in group E, which weakly increased the ratio of luciferase activity, are hardly expressed in human liver (Table S3).

### SOX7 directly binds to the functional SNP

**Figure 3:**
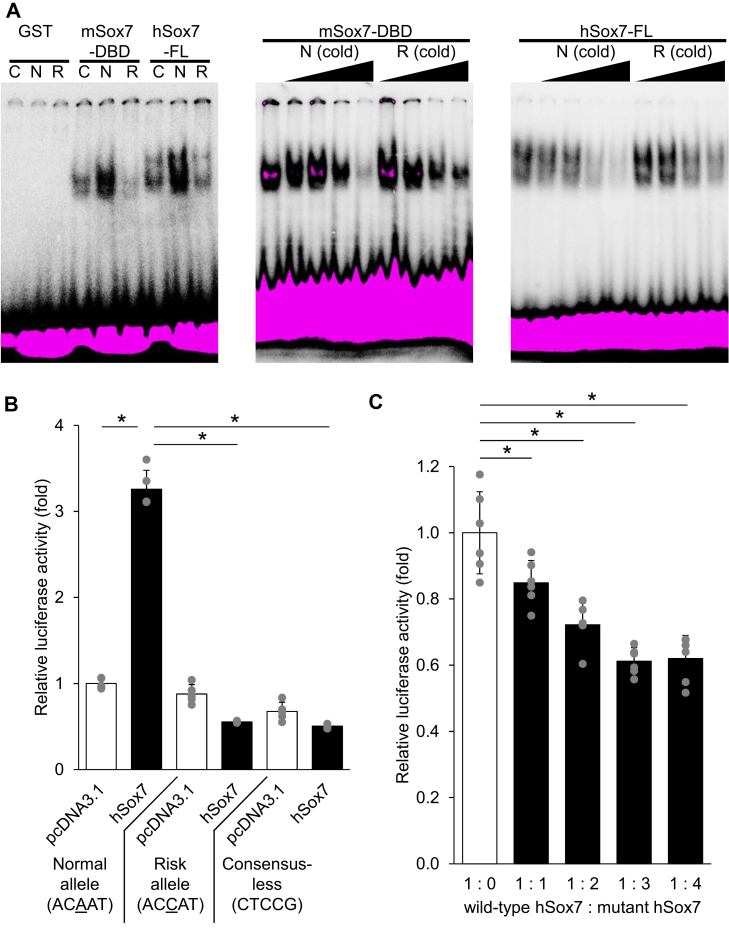
**Sox7 directly binds to rs12718466 in the APOA1 promoter.***A*, *Left*: the DNA-binding domain of mouse Sox7 (mSox7-DBD) and the full-length human Sox7 (hSox7-FL) were used as glutathione S-transferase (GST) fusion proteins. Control probe (C), normal allele probe (N), and risk allele probe (R) contained the Sox7 consensus sequence, the rs12718466 normal allele (adenine), and the rs12718466 risk allele (cytosine), respectively. *Center*: effects of cold probes on the binding of the mSox7-DBD to the control probe. *Right*: effects of cold probes on the binding of the hSox7-FL to the normal allele probe. *B*, the APOA1 promoter without any SNP, with only the rs12718466 risk allele (cytosine), and with the substituted Sox7consensus sequence respectively were inserted into firefly luciferase reporter plasmid. These plasmids were cotransfected with the hSox7 expression plasmid and pRL-SV40 into HEK293 cells, and luciferase activity was measured (n = 5). Data are expressed as means ± SD. Significant differences were assessed using the unpaired two-tailed Student's *t* test. The differences were considered to be significant if *p* < 0.05. *C*, the APOA1 promoter without any SNP was inserted into firefly luciferase reporter plasmid. In addition to the hSox7, a human dominant-negative mutant lacking the transactivation domain was used as expression plasmids. These plasmids were cotransfected with firefly luciferase reporter plasmid and pRL-SV40 into HEK293 cells, and luciferase activity was measured (n = 6). Data are expressed as means ± SD. Significant differences were assessed using ANOVA, when indicated by appropriate *p* values (*p* < 0.05), by Dunnett *post hoc* test. The differences were considered to be significant if *p* < 0.05. APOA1, apolipoprotein A1; SOX7, SRY-box transcription factor 7.

Electrophoresis mobility shift assay (EMSA) was performed to qualitatively demonstrate allele-dependent Sox7-DNA binding and competitive displacement; the assay’s nonequilibrium/condition sensitivity precludes reliable determination of absolute Kd under our conditions. For clarity, we denote mouse Sox7 as mSox7 and human Sox7 as hSox7 throughout the manuscript. As previously reported (15), GST-mSox7-DBD bound to the control probe containing the Sox7 consensus sequence (Fig. 3*A*). GST-hSox7-FL also bound to the control probe. Both GST-mSox7-DBD and GST-hSox7-FL robustly bound to the normal allele probe, whereas these GST fusion proteins bound weakly to the risk allele probe. Binding of these GST fusion proteins to the normal allele probe was attenuated in a dose-dependent manner by the addition of a cold probe containing the normal allele; however, attenuation was less pronounced with a cold probe containing the risk allele.

The effect of the normal and risk alleles on transcriptional activity was evaluated using a luciferase assay with reporter plasmids containing three different APOA1 promoter sequences (Fig. 3*B*). In APOA1 pro-Firefly Luc, hSox7 significantly increased luciferase activity, whereas in the reporter plasmid with risk allele, hSox7 did not increase luciferase activity. hSox7 also did not increase luciferase activity in the reporter plasmid where the Sox7 consensus sequence was substituted by mutagenesis. Competitive inhibition experiments using dominant-negative mutants revealed that Sox7 directly binds to rs12718466. As shown in Figure 3*C*, the increase in luciferase activity resulting from hSox7 binding to the cis-element of APOA1 pro-Firefly Luc was reduced in a dose-dependent manner by the addition of mutant Sox7. In this assay, dominant-negative Sox7 is expected to bind DNA but lacks transactivation capacity, thereby competing with full-length Sox7 for the rs12718466-containing element; the observed dose-dependent reduction in reporter activation is consistent with this dominant-negative mechanism.

### Knockdown of SOX7 decreases APOA1 gene expression and plasma HDL-C levels

**Figure 4:**
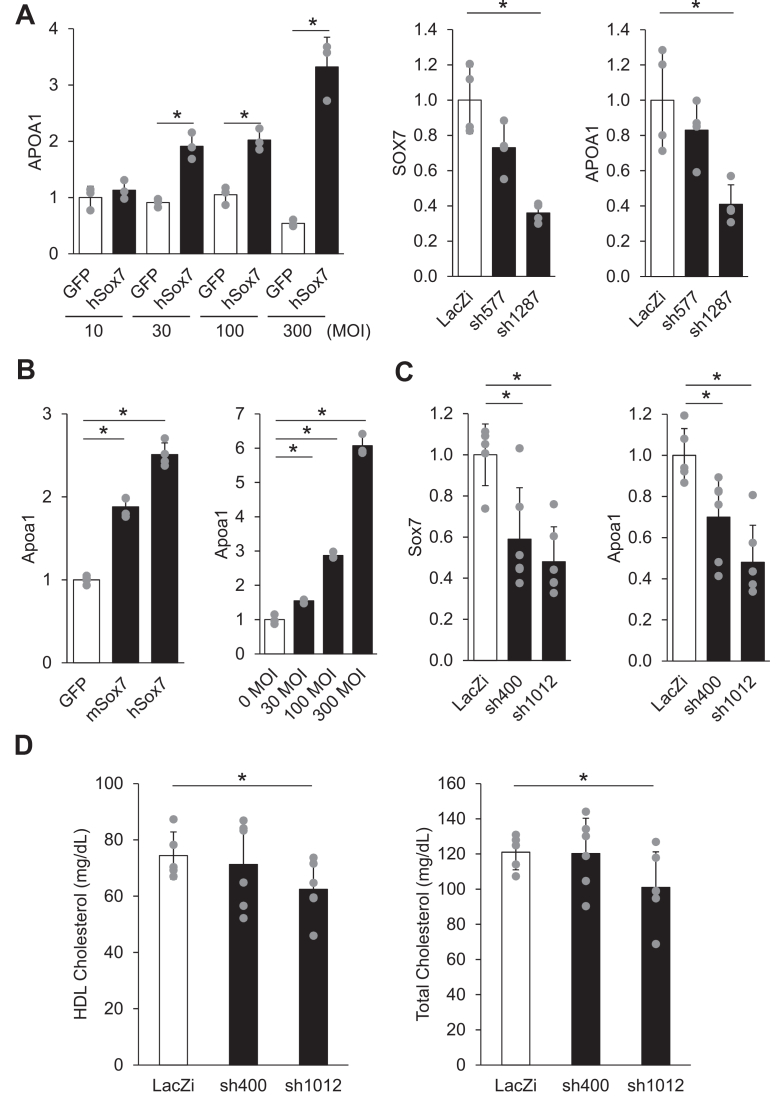
**Overexpression and knockdown of Sox7 affect APOA1 gene expression and HDL-C levels.***A*, *Left*: overexpression of Sox7 in HepG2 cells. Adenoviruses expressing either GFP or hSox7 were infected at the indicated MOI in HepG2 cells. RNA was extracted 4 days after infection, and endogenous APOA1 gene expression was quantified by quantitative reverse transcription PCR (n = 3). Data are expressed as means ± SD. Significant differences were assessed using the unpaired two-tailed Student's *t* test. The differences were considered to be significant if *p* < 0.05. *Right*: knockdown of Sox7 in HepG2 cells. Adenoviruses expressing shRNA targeting either LacZ or hSox7 were infected at 100 MOI in HepG2 cells. RNA was extracted 4 days after infection, and endogenous SOX7 and APOA1 gene expression were quantified by quantitative reverse transcription PCR (n = 4). Data are expressed as means ± SD. Significant differences were assessed using ANOVA, when indicated by appropriate *p* values (*p* < 0.05), by Dunnett *post hoc* test. The differences were considered to be significant if *p* < 0.05. *B*, *Left*: primary hepatocytes were infected with adenoviruses expressing GFP, mSox7, or hSox7 at 30 MOI. RNA was extracted 2 days after infection, and endogenous Apoa1 gene expression was quantified by quantitative reverse transcription PCR (n = 4). Data are expressed as means ± SD. Significant differences were assessed using ANOVA, when indicated by appropriate *p* values (*p* < 0.05), by Dunnett *post hoc* test. The differences were considered to be significant if *p* < 0.05. *Right*: primary hepatocytes were infected with adenoviruses expressing hSox7 at the indicated MOI. RNA was extracted 2 days after infection, and endogenous Apoa1 gene expression was quantified by quantitative reverse transcription PCR (n = 3). Data are expressed as means ± SD. Significant differences were assessed using ANOVA, when indicated by appropriate *p* values (*p* < 0.05), by Dunnett *post hoc* test. The differences were considered to be significant if *p* < 0.05. *C*, adenoviruses expressing shRNA targeting either LacZ or mSox7 were infected in five-week-old ICR mice. Five days after infection, livers were collected for RNA extraction to quantify endogenous Sox7 and Apoa1 expression (n = 5–6). Data are expressed as means ± SD. Significant differences were assessed using ANOVA, when indicated by appropriate *p* values (*p* < 0.05), by Dunnett *post hoc* test. The differences were considered to be significant if *p* < 0.05. *D*, adenoviruses expressing shRNA targeting either LacZ or mSox7 were infected in five-week-old ICR mice. Five days after infection, blood samples were collected at necropsy to measure HDL-C and total cholesterol levels (n = 5–6). Data are expressed as means ± SD. Significant differences were assessed using the unpaired one-tailed Student's *t* test. The differences were considered to be significant if *p* < 0.05. APOA1, apolipoprotein A1; HDL-C, high-density lipoprotein cholesterol; MOI, multiplicity of infection; SOX7, SRY-box transcription factor 7.

To investigate the effect of Sox7 on APOA1 gene expression, we first performed overexpression and knockdown experiments in culture cells. HepG2 cells are known to have higher endogenous APOA1 gene expression than Hep3B cells, and we confirmed this by quantitative PCR (Fig. S2). We also sequenced the HepG2 cell genome to confirm that rs12718466 was the normal allele (data not shown). As shown in Figure 4*A*, Ad-hSox7 significantly increased APOA1 gene expression in a dose-dependent manner. Conversely, suppression of Sox7 with shRNA significantly decreased APOA1 gene expression in HepG2 cells.

Next, we conducted overexpression experiments in primary hepatocytes. Sox7 significantly increased Apoa1 gene expression when primary hepatocytes were infected with Ad-GFP, Ad-mSox7, or Ad-hSox7 at 30 multiplicity of infection (MOI) (Fig. 4*B*). Ad-hSox7 also increased Apoa1 gene expression significantly in a dose-dependent manner from 30 to 300 MOI.

Finally, we investigated whether knocking down Sox7 decreases Apoa1 gene expression and reduces HDL-C levels in mice. Mice were infected with adenoviruses expressing either an shRNA targeting Sox7 or a control shRNA targeting LacZ. Gene expression in the liver and HDL-C levels in the blood were then assessed 5 days later. Knockdown of Sox7 in mice was found to significantly decrease Apoa1 gene expression (Fig. 4*C*) and reduce both HDL-C and TC levels (Fig. 4*D*).

## Discussion

In this study, we identified rs12718466 as the functional SNP mapping to the transcriptional regulatory region of the APOA1 gene. In addition, we demonstrated that this noncoding SNP interacts with the transcription factor SOX7 to regulate APOA1 gene expression and plasma HDL-C levels.

A prevailing view of the atheroprotective effect of HDL is that it stimulates the reverse transport of cholesterol from arterial lesions to the liver (16). The overexpression of APOA1 in apoE-deficient mice (17) and western diet-fed low-density lipoprotein receptor-deficient mice (18) limited the progression of fatty streak lesions and led to lesion regression. Therefore it is quite reasonable to search for genetic factors that determine blood HDL levels in the APOA1 gene, but identifying functional SNPs among noncoding SNPs rather than missense mutations is challenging and has not been thoroughly explored so far.

Our experiments identified rs12718466 as the noncoding functional SNP among 35 candidates upstream of the APOA1 gene that may influence APOA1 gene expression in the liver (Fig. 1*B*). Consistent with this finding, available GTEx liver data (https://platform.opentargets.org/study/gtex_tx_liver_enst00000236850) show that the alternative allele reduces APOA1 expression in the liver with a normalized effect size of −1.47 (*p* = 1.25e-17) (19).

The SNP rs12718466 is located 325 bp upstream of the TSS of the human APOA1 gene. Some earlier studies have suggested that the transcriptional regulatory elements are located 192 to 256 bp (20) (199–249 bp (21)) upstream of the TSS in HepG2 cells. However, no consensus has been reached regarding the region further upstream. Although subsequent studies of this region have been insufficient, our study was consistent with the finding that some transcriptional regulatory activity had been observed (21). In fact, JASPAR (https://jaspar.elixir.no/), a database of transcription factor binding profiles, predicted that multiple transcription factors, including SOX proteins, presumably bind to rs12718466 (data not shown) (22).

We previously described the development of a TFEL covering nearly all transcription factors in the mouse genome (8). Using this library, we established a new screening approach called the TFEL scan method, which allows identification of upstream transcription factors regulating specific promoters or enhancers in a chromosomal context-dependent manner (9, 23, 24). The TFEL scan method provides a powerful, systematic tool to identify upstream transcription factors and their cooperative networks. It enables exploration of promoter- and enhancer-specific regulation in a physiologically relevant context, offering new opportunities for mapping transcriptional regulatory networks.

We demonstrated that SOX7 significantly increased APOA1 gene expression in HepG2 cells and primary hepatocytes. Conversely, suppression of Sox7 with shRNA significantly decreased APOA1 gene expression in HepG2 cells and mouse livers (Fig. 4, *A–C*). SOX7 belongs to the SOX (SRY-related HMG-box) family of transcription factors that have been shown to regulate multiple biological processes, such as hematopoiesis, vasculogenesis, and cardiogenesis during embryonic development (25). However, relatively little has been known about the role of SOX7 in the adult liver, because the homozygous deletion of SOX7 results in embryonic lethality before embryonic day 14.5 due to cardiovascular failure (26). Previous reports have suggested that SOX7 may play a role in maintaining normal liver function. The study investigating the potential role of SOX genes in hepatocellular carcinoma revealed that Sox7 expression significantly decreased in human hepatocellular carcinoma tissues and Sox18 expression significantly increased, whereas Sox17 expression was not significantly altered (27). The SOX7 expression level in liver cancer tissues was significantly lower than that observed in noncancerous liver tissues. Immunohistochemistry revealed that strong SOX7 staining was observed in the adjacent noncancerous liver tissues (28). Interestingly, 13 genes including SOX7 showed reduced expression in human fatty liver and contained a conserved binding motif for microRNA-21 (29). In parallel, hepatocyte-derived APOA1 is not only the major HDL apolipoprotein but also exerts hepatoprotective actions; in mice, ApoA1 accelerates liver regeneration *via* AMPK-ULK1-dependent autophagy (30). Thus, SOX7 may contribute to maintaining liver function by regulating APOA1 gene expression.

Knockdown of Sox7 in mice decreased ApoA1 gene expression and consistently reduced HDL-C and TC levels (Fig. 4, *C* and *D*). Interestingly, when evaluating the relationship between circulating lipoprotein lipids and apolipoproteins with risk of coronary heart disease, a lead SNP rs1986868 located approximately 10 kb upstream of SOX7 was found to be associated with reduced APOA1 levels, which is consistent with our results (31).

EMSA and luciferase assay as well as overexpression/knockdown experiments support an allele-dependent role of SOX7 at rs12718466. Although precise single-nucleotide editing in hepatocyte-derived cells remains technically challenging due to limited homology-directed repair efficiency and the practical difficulty of clonal isolation, a gold-standard test would be CRISPR knock-in of the rs12718466 risk allele in these cells to assess APOA1 regulation in its native chromatin context. We therefore regard allele-specific knock-in and subsequent phenotypic assessment as a key future direction to further strengthen the mechanistic inference.

Overall, using the TFEL scan method, we demonstrate that rs12718466 is the noncoding functional SNP that alters APOA1 gene expression by interacting with the transcription factor SOX7 in hepatocytes.

## Experimental procedures

### Search for candidate functional SNPs

According to ChIP-Atlas (https://chip-atlas.org/), the CTCF binding site upstream of the APOA1 gene is located near chr11:116840500 (hg38), and there are no CTCF binding sites from there to the APOA1 gene body. On the other hand, several isoforms are transcribed from the APOA1 gene, and these isoforms share the same exon 2. Based on these data, the search range for functional SNPs was set to a genomic region that included the CTCF binding site on the 5′ side and excluded exon 2 on the 3′ side. To clone this genomic region (chr11:116837408–116840654), weused the following primers: promoter-MluI-F 5′-ACGCGTTCAGCTCTGTCCAGAAAGACCT-3′ and promoter-BamHI-R 5′-GGATCCCTGGAGGAGAAGAAGGGCCT-3’. After confirming that the sequence was identical to the reference sequence by sequencing, the fragment was inserted into the MluI and BglII sites of pGL3 basic plasmid (Promega Corporation) (APOA1 pro-Firefly Luc). Similarly, the fragment was inserted into a modified pGL3 basic plasmid whose firefly luciferase reporter gene was substituted by renilla luciferase reporter gene (APOA1 pro-Renilla Luc).

The dbSNP (Build 138) identified 62 SNPs in this cloned genomic region (3247 bp). Among these SNPs, 14 were common SNPs, which had a MAF of more than 0.01 (Table S1). In addition to the common SNPs, this study also targeted rare SNPs, which had at least one linkage SNP with r^2^ greater than 0.8 in African, American, Asian, or European populations, using a search space of all variants within 250 kilobases of each other. According to HaploReg (https://pubs.broadinstitute.org/mammals/haploreg/haploreg.php), twenty-one rare SNPs fulfilled the criteria (Table S1). Genomic regions that were confirmed to be consistent with the reference sequence were substituted with each SNP and then inserted into pGL3 basic plasmid. The mutated fragments were generated using PrimeSTAR Mutagenesis Basal Kit (Takara Bio Inc) based on PCR according to the mutation method (32). For rs12691374, two types of risk alleles were known, so both variants were generated.

### Cell culture

HEK293 human embryonic kidney cells (RRID:CVCL_0045), HepG2 human hepatoma cells (RRID:CVCL_0027), and Hep3B human hepatoma cells (RRID:CVCL_0326) were distributed from the American Type Culture Collection (ATCC), and cultured in Dulbecco's modified Eagle's medium (DMEM) containing 25 mm glucose, 100 U·mL^−1^ penicillin, and 100 μg·mL^−1^ streptomycin sulfate supplemented with 10% fetal bovine serum. AML12 mouse hepatocytes (RRID:CVCL_0140) were distributed from ATCC, and cultured in DMEM/F12 containing ITS-G Supplement (Wako Pure Chemical Industries), 100 U·mL^−1^ penicillin, and 100 μg·mL^−1^ streptomycin sulfate supplemented with 10% fetal bovine serum. All cell lines have been authenticated by our facility’s authentication protocol within the last 3 years, and all experiments were performed with mycoplasma-free cells (32).

### Luciferase assay

The indicated expression plasmids, firefly luciferase reporter plasmid, and renilla luciferase reporter plasmid were cotransfected into cells using SuperFect Transfection Reagent (QIAGEN) according to the manufacturer’s protocol. Unless otherwise noted, pRL-SV40 (Promega Corporation) was used as the renilla luciferase reporter plasmid. Total amounts of transfected DNA were adjusted with empty plasmid. The luciferase activity in transfectant was measured on a luminometer as described previously (9).

### Genome-wide transcription factor screen

As we previously described (9), a 25-bp DNA fragment centered on the identified functional SNP was inserted into a reporter plasmid to enhance the sensitivity of the transcription factor screen. The fragment of three tandem copies of the 25-bp region containing the risk allele of rs12718466 (Table S2) was cloned into the BamHI and SalI sites located at the 3′ end of the firefly luciferase reporter gene of APOA1 pro-Firefly Luc (APOA1 pro-Firefly Luc-rs12718466x3). Similarly, the fragment of three tandem copies of the 25-bp region containing the normal allele (Table S2) was cloned at the 3′ end of the renilla luciferase reporter gene of APOA1 pro-Renilla Luc (APOA1 pro-Renilla Luc-rs12718466x3). For the primary TFEL screen, pools of 10 transcription factors were assembled in a randomized manner from our in-house expression library (8). No grouping by structural class (*e.g.*, zinc finger, homeodomain, and HMG-box) or predicted function was used. This unbiased randomization was adopted to avoid structural/functional enrichment, thereby enabling a comprehensive and neutral survey of the transcription-factor repertoire. In the secondary screen, transfections of single transcription factor were performed for all candidates emerging from the positive pools of 10 transcription factors. HEK293 cells in a 48-well plate were cotransfected with the TFEL clones (8) and reporter plasmids mentioned above. Cells were lysed in Reporter Lysis Buffer (Promega Corporation) 24 h after transfection, and then firefly and renilla luciferase activities were measured (9).

### Phylogenetic tree

The Sox family tree displayed in Figure 2*C* was adapted from the published HMG-domain phylogeny of Sox proteins reported by Kamachi and Kondoh (13). For the present study, we updated the layout and annotations (*e.g.*, labels consistent with the TF groups analyzed here) to improve readability and contextual alignment with our data; no *de novo* computational reconstruction (sequence re-selection, re-alignment, or re-estimation) was carried out.

### Electrophoretic mobility shift assay

To synthesize the glutathione S-transferase (GST) fusion protein of mouse Sox7 DNA binding domain (mSox7-DBD), ranging from 37 to 131 amino acid residues, the fragment was inserted into the SmaI site of pGEX-4T-2 (GE Healthcare) using the following primers: mSox7binding-GST-F 5′-GGGGACAAGAGTTCGGAAAG-3’; mSox7binding-Stop-R 5′-AGGGTCCACGCGCTTGCA-3’ (15). Similarly, for the GST fusion protein of full-length human Sox7 (hSox7-FL), the fragment was inserted into the BamHI and NotI sites of pGEX-4T-2 using the following primers: hSox7-GST-BamHI-F 5′-GGATCCATGGCTTCGCTGCTGGGAGCC-3’; hSox7-Stop-NotI-R 5′-GCGGCCGCCTATGACACACTGTAGCTGTTGTAGTACGTGGCC-3’. GST and GST fusion proteins were prepared as described previously (23) and dialyzed with dialysis buffer (50 mM Tris–HCl [pH 8.0], 50 mM NaCl, 1 mM EDTA, and 1 mM DTT). EMSA was performed as previously described (9). The sequences of the probes used were as follows: control probe (15): AGCTTATAACAATGATCCACGAA; normal allele probe: GCCAACACAATGGACAATGGCAAC; risk allele probe: GCCAACACCATGGACAATGGCAAC. The probes were labeled with [α-32P] dCTP by filling in the 5′-overhangs with Klenow DNA polymerase (GE HealthCare) and purified on Sephadex G-25 (GE HealthCare) columns. The labeled DNA probe was incubated with GST and GST fusion proteins in a buffer containing 10 mM Hepes at pH 7.8, 50 mM KCl, 1 mM EDTA, 5 mM MgCl_2_, 5 mM dithiothreitol, 30 μg mL^−1^ poly(dI-dC), and 0.1% Triton X-100, for 30 min on ice. The protein complex was resolved on 4.6% polyacrylamide gels in 1xTBE buffer.

## Construction of expression plasmids

The expression plasmid of human Sox7 was constructed using the following primers: hSox7-HA-BamHI-F 5′-GGATCCGCCACCATGTACCCATACGATGTTCCAGATTACGCTATGGCTTCGCTGCTGGGAGCC-3’; Reverse primer had the same sequence as hSox7-Stop-NotI-R mentioned above. Similarly, the expression plasmid of mouse Sox7 was constructed using the following primers: mSox7-HA-EcoRI-F 5′-GAATTCGCCACCATGTACCCATACGATGTTCCAGATTACGCTATGGCCTCGCTGCTGGGCG-3’; mSox7-Stop-XbaI-R 5′-TCTAGACTATGACACACTGTAGCTGTTGTAATACGTGGCCG-3’. After amplification by PCR, the products were cloned into pGEM-T easy (Promega Corporation). The complementary DNA (cDNA) fragments were cut out from the plasmid and ligated into the indicated restriction enzyme sites of pcDNA3.1(+) (Invitrogen). Using the mouse Sox7 truncated construct (33) as a reference, we generated a human dominant-negative mutant that had a DNA binding domain but no transactivation domain. The dominant-negative Sox7 was designed to delete the C terminal transactivation domain while retaining the N terminal HMG-box DNA-binding domain (which mediates minor-groove binding and DNA bending characteristic of Sox proteins). This modular organization and the use of transactivation-deficient Sox constructs as competitive inhibitors are well established for Sox family members; thus our design follows validated principles and the previously reported Sox7 truncation map (33). The fragment was cut out by BamHI and ScaI from the expression plasmid of human Sox7 and ligated into the BamHI and EcoRV sites of pcDNA3.1(+).

### Preparation and transduction of recombinant adenoviruses

The fragments containing human and mouse Sox7 cut from each expression plasmid were inserted into the Gateway entry vector pENTR4 (Invitrogen), and then the adenoviral plasmids were generated by homologous recombination between the entry vector and the pAd/CMV/V5-DEST vector (Invitrogen). After the transfection of the plasmid into HEK293 cells, recombinant adenoviruses were collected by CsCl gradient centrifugation as described previously (9). Adenovirus encoding GFP (Ad-GFP) was described previously (23).

Human Sox7-specific shRNA expression plasmids were cloned into pENTR/U6 entry vector (Invitrogen). The target sequences of shRNA, named based on the position at the beginning of each target sequence in the mRNA, were as follows: sh577 5′-CCGGAGAAGAGAAGCGGCA-3’; sh1287 5′-CAACAGCTACAGTGTGTCA-3’. Similarly, mouse Sox7-specific shRNA expression plasmids were cloned using the following target sequences: sh400 5′-GCAGGAAGAAACAAGGCAA-3’; sh1012 5′-GCAATGAATTTGATCAGTA-3’. Adenovirus vectors for knockdown were generated by homologous recombination between the entry vector and the pAd promoterless vector using the Gateway system (9). LacZ-specific shRNA expression plasmids (LacZi) and adenovirus vector (Ad-LacZi) were described previously (23).

The titer of adenoviruses was determined using the Adeno-X Rapid Titer Kit (Clontech Laboratories). For overexpression experiments, adenoviruses were infected at the indicated MOI in HepG2 cells; for knockdown experiments, adenoviruses were infected at 100 MOI in HepG2 cells. Four days after infection, RNA preparation was performed. Primary hepatocytes were isolated from mice with collagenase perfusion method as described previously (24). Adenoviruses were infected at 30 to 300 MOI in primary hepatocytes. Two days after infection, RNA preparation was performed.

Five-week-old ICR male mice were purchased from SLC. All animals were maintained in a temperature-controlled environment with a 14 h-light/10 h-dark cycle and were given free access to the standard laboratory diet and water. All animals studied were anesthetized and euthanized according to the protocol approved by the Tsukuba University Animal Care and Use Committee (9). Adenoviruses were injected intravenously into ICR male mice from subclavian vein at the following doses: for Ad-LacZi, Ad-sh400, and Ad-sh1012, 1 × 10^9^ PFU, and 1000 optical particles of adenovirus were calculated as 1 PFU. Five days after infection, RNA preparation was performed.

### RNA isolation and quantitative reverse transcription PCR

Total RNA was extracted with Sepasol reagent (Nacalai Tesque) and transcribed into cDNA using reverse transcription reagent kit (TOYOBO). Quantitative reverse transcription PCR was performed using ABI 7500 System (Applied Biosystems 7500 Fast, Applied Biosystems). Quantitative reverse transcription PCR was performed using SYBR Green PCR master mix (9). The primer sets were as follows: ACTB, 5′-TCGTGCGTGACATTAAGGAG-3′ and 5′-ATGCCAGGGTACATGGTGGT-3’ (34); SOX7, 5′-ACCAACGGGTCCCACAGA-3′ and 5′-CCACTCAAGGCACAAGAAGG-3’ (34); APOA1, 5′-AGACAGCGGCAGAGACTATGTGT-3′ and 5′-CCAGTTGTCAAGGAGCTTTAGGTT-3’ (35); Actb, 5′-AGCCATGTACGTAGCCATCCA-3′ and 5′-TCTCCGGAGTCCATCACAATG-3’ (36); Sox7, 5′-CAGCAAGATGCTGGGAAAGT-3′ and 5′-GGCCGGTACTTGTAGTTGGG-3’; Apoa1, 5′-CTTGGCACGTATGGCAGCA-3′ and 5′-CCAGAAGTCCCGAGTCAATGG-3’ (37).

### Blood tests

HDL-C and TC levels were measured using the HDL Cholesterol E-test Wako (Wako Pure Chemical Industries) or the Cholesterol E-test Wako (Wako Pure Chemical Industries) according to the manufacturer’s protocol.

### Statistical analyses

Data are expressed as means ± SD. Individual data points indicated independent biological replicates. Differences between two groups were assessed using the unpaired two-tailed Student’s *t* test, unless otherwise specified. Datasets involving more than two groups were assessed using ANOVA, when indicated by appropriate *p* values (*p* < 0.05), by Dunnett *post hoc* test, with StatView Software (BrainPower). The differences were considered to be significant if *p* < 0.05.

## Data availability

All data supporting the findings of this study are available within the article and its supporting information files.

## Supporting information

This article contains supporting information (10,14,38,39).

## Conflict of interest

The authors declare that they have no conflicts of interest with the contents of this article.

## References

[1] Virani S.S., Newby L.K., Arnold S.V., Bittner V., Brewer L.C., Demeter S.H. (2023). Circulation.

[2] Teslovich T.M., Musunuru K., Smith A.V., Edmondson A.C., Stylianou I.M., Koseki M. (2010). Nature.

[3] Rosenson R.S., Brewer H.B., Ansell B.J., Barter P., Chapman M.J., Heinecke J.W. (2016). Nat. Rev. Cardiol..

[4] Kanai M., Akiyama M., Takahashi A., Matoba N., Momozawa Y., Ikeda M. (2018). Nat. Genet..

[5] Sinnott-Armstrong N., Tanigawa Y., Amar D., Mars N., Benner C., Aguirre M. (2021). Nat. Genet..

[6] Barton A.R., Sherman M.A., Mukamel R.E., Loh P.R. (2021). Nat. Genet..

[7] Alsheikh A.J., Wollenhaupt S., King E.A., Reeb J., Ghosh S., Stolzenburg L.R. (2022). BMC Med. Genomics.

[8] Yahagi N., Takeuchi Y. (2021). F1000Res.

[9] Piao X., Yahagi N., Takeuchi Y., Aita Y., Murayama Y., Sawada Y. (2018). FEBS Lett..

[10] Wang Y., Song F., Zhang B., Zhang L., Xu J., Kuang D. (2018). Genome Biol..

[11] Mishiro T., Ishihara K., Hino S., Tsutsumi S., Aburatani H., Shirahige K. (2009). Embo j.

[12] Ward L.D., Kellis M. (2016). Nucleic Acids Res..

[13] Kamachi Y., Kondoh H. (2013). Development.

[14] Ono H., Ogasawara O., Okubo K., Bono H. (2017). Sci. Data.

[15] Taniguchi K., Hiraoka Y., Ogawa M., Sakai Y., Kido S., Aiso S. (1999). Biochim. Biophys. Acta.

[16] Tall A.R., Jiang X., Luo Y., Silver D. (2000). Arterioscler Thromb. Vasc. Biol..

[17] Benoit P., Emmanuel F., Caillaud J.M., Bassinet L., Castro G., Gallix P. (1999). Circulation.

[18] Tangirala R.K., Tsukamoto K., Chun S.H., Usher D., Pure E., Rader D.J. (1999). Circulation.

[19] Consortium G.T. (2020). Science.

[20] Sastry K.N., Seedorf U., Karathanasis S.K. (1988). Mol. Cell Biol.

[21] Higuchi K., Law S.W., Hoeg J.M., Schumacher U.K., Meglin N., Brewer H.B. (1988). J. Biol. Chem..

[22] Rauluseviciute I., Riudavets-Puig R., Blanc-Mathieu R., Castro-Mondragon J.A., Ferenc K., Kumar V. (2024). Nucleic Acids Res..

[23] Takeuchi Y., Yahagi N., Aita Y., Murayama Y., Sawada Y., Piao X. (2016). Cell Rep.

[24] Takeuchi Y., Yahagi N., Aita Y., Mehrazad-Saber Z., Ho M.H., Huyan Y. (2021). iScience.

[25] Stovall D.B., Cao P., Sui G. (2014). Histol histopathol.

[26] Wat M.J., Beck T.F., Hernandez-Garcia A., Yu Z., Veenma D., Garcia M. (2012). Hum. Mol. Genet..

[27] Huang W., Chen Z., Shang X., Tian D., Wang D., Wu K. (2015). Hepatology.

[28] Wang J., Zhang S., Wu J., Lu Z., Yang J., Wu H. (2017). Mol. Med. Rep..

[29] Wu H., Ng R., Chen X., Steer C.J., Song G. (2016). Gut.

[30] Wang Z.Y., Chen R.X., Wang J.F., Liu S.C., Xu X., Zhou T. (2024). Cell Mol Gastroenterol Hepatol.

[31] Richardson T.G., Sanderson E., Palmer T.M., Ala-Korpela M., Ference B.A., Smith G.D. (2020). et al.Evaluating the relationship between circulating lipoprotein lipids and apolipoproteins with risk of coronary heart disease: a multivariable Mendelian randomisation analysis. PLoS Med.

[32] Takeuchi Y., Murayama Y., Aita Y., Mehrazad Saber Z., Karkoutly S., Tao D. (2024). FEBS J.

[33] Takash W., Cañizares J., Bonneaud N., Poulat F., Mattéi M.G., Jay P. (2001). Nucleic Acids Res..

[34] Zhang Y., Huang S., Dong W., Li L., Feng Y., Pan L. (2009). Cancer Lett..

[35] Arishima H., Matsunaga A., Uehara Y., Niimura H., Zhang B., Ohwaki K. (2011). Med. Bull. Fukuoka Univ..

[36] Grefhorst A., Elzinga B.M., Voshol P.J., Plösch T., Kok T., Bloks V.W. (2002). J. Biol. Chem..

[37] Tao H.C., Chen K.X., Wang X., Chen B., Zhao W.O., Zheng Y. (2020). Front. Immunol..

[38] Sherry S.T., Ward M.-H., Kholodov M., Baker J., Phan L., Smigielski E.M. (2001). dbSNP: the NCBI database of genetic variation. Nucleic Acids Res..

[39] Brown G.R., Hem V., Katz K.S., Ovetsky M., Wallin C., Ermolaeva O. (2015). et al.Gene: a gene-centered information resource at NCBI. Nucleic Acids Res..

