# Supplementary material for: A functional SNP rs12718466 in APOA1 promoter modulates gene expression via interaction with SOX7
Source: J Biol Chem. 2026 Apr 27;302(6):113101. doi: 10.1016/j.jbc.2026.113101 (PMC13226946; doi:10.1016/j.jbc.2026.113101)
Supplement: Supporting Information [file mmc1.pdf]

Supporting Information

A functional SNP rs12718466 in APOA1 promoter modulates gene expression via interaction with SOX7

Yuichi Aita,[1,2] Yoshinori Takeuchi,[1] Yukari Masuda,[1] Zahra Mehrazad Saber,[1] Samia Karkoutly,[1] Duhan Tao,[1,2] Chen Ye,[1,2] Tsolmon Mendsaikhan,[1] Rika Saikawa,[1] Yuki Murayama,[2,3] Akito Shikama,[2,3] Takashi Matsuzaka,[3] Hitoshi Shimano,[3] Yasushi Kawakami,[3] and Naoya Yahagi.[1,2,3]

From
Division of Endocrinology and Metabolism,
Department of Medicine, Jichi Medical University, Tochigi 329-0498, Japan[1]
Nutrigenomics Research Group, Institute of Medicine, University of Tsukuba, Ibaraki 305-8575, Japan[2]
Department of Internal Medicine (Endocrinology and Metabolism), Institute of Medicine, University of Tsukuba, Ibaraki 305-8575, Japan[3]

Table S1. Thirty-five candidate functional SNPs upstream of the APOA1 gene.
Table S2. The fragment of three tandem copies of the 25-bp region containing the 'normal allele' and 'risk allele' of rs12718466.
Table S3. Database information on the expression of Sox proteins in human liver.
Figure S1. Apolipoprotein genes form one topologically associating domain on human chromosome 11.
Figure S2. Endogenous APOA1 gene expression in HepG2, Hep3B, and HEK293 cells.
Figure S3. Reporter-based screen in AML12, a mouse hepatocyte cell line.
Figure S4. shRNA knockdown efficiency against hSox7 and mSox7.

Table S1. Thirty-five candidate functional SNPs upstream of the APOA1 gene.

Position (hg38)	SNP ID	MAF (1000Genomes)	Common/Rare
11:116840634	rs11216158	0.216653	Common
11:116840464	rs11216157	0.219649	Common
11:116840425	rs2727784	0.34385	Common
11:116840367	rs187089134	0.002596	
11:116840347	rs181696828	0.0002	
11:116840334	rs151155090	0.002796	
11:116840277	rs111255936	0.205471	Common
11:116840252	rs613808	0.36222	Common
11:116840206	rs142098595	0.000599	
11:116840133	rs147608509	0.000599	
11:116840066	rs145254627	0.008986	
11:116840022	rs148473539	0.000998	
11:116839976	rs189016737	0.000998	
11:116839827	rs149971793	0.0002	
11:116839523	rs632153	0.06889	Common
11:116839451	rs4018880	0.428315	Common
11:116839334	rs184323875	0.000799	
11:116839299	rs191775753	0.002396	
11:116839130	rs146111781	0.003195	
11:116838722	rs1374117	0.181709	Common
11:116838681	rs184514487	0.000399	
11:116838667	rs148154120	0.000998	
11:116838651	rs143816409	0.0002	
11:116838616	rs149441358	0.002396	
11:116838524	rs192194271	0.000399	
11:116838385	rs45589834	0.001597	
11:116838320	rs138372742	0.003195	
11:116838309	rs112198691	0.018171	Common
11:116838241	rs142031918	0.004593	
11:116838225	rs147593546	0.0002	
11:116838061	rs12691374	0.01797	Common
11:116837986	rs12718467	0.032149	Common
11:116837947	rs12718466	0.028554	Common
11:116837697	rs670	0.188498	Common
11:116837538	rs5069	0.121805	Common

This information was referenced from dbSNP (https://www.ncbi.nlm.nih.gov/snp/).

Table S2. The fragment of three tandem copies of the 25-bp region containing the ‘normal allele’ and ‘risk allele’ of rs12718466.

rs12718466 Normal allele (3X)	
sense (5'-3')	GATCCCTCTGCCAACACAATGGACAATGGCCTCTGCCAACACAATGGACAATGGCCTCTGCCAACACAATGGACAATGGCG
antisense (5'-3')	TCGACGCCATTGTCCATTGTGTTGGCAGAGGCCATTGTCCATTGTGTTGGCAGAGGCCATTGTCCATTGTGTTGGCAGAGG
rs12718466 Risk allele (3X)	
sense (5'-3')	GATCCCTCTGCCAACACCATGGACAATGGCCTCTGCCAACACCATGGACAATGGCCTCTGCCAACACCATGGACAATGGCG
antisense (5'-3')	TCGACGCCATTGTCCATGGTGTTGGCAGAGGCCATTGTCCATGGTGTTGGCAGAGGCCATTGTCCATGGTGTTGGCAGAGG

Table S3. Database information on the expression of Sox proteins in human liver.

	HPA RNA-seq normal tissues (RPKM)*	CAGE (cap analysis of gene expression) (TPM)**	RNA-seq (FPKM)**
Group E			
SOX8	0	0	0.03
SOX9	1.464±0.515	2.12	1.00
SOX10	0	0.36	0.10
Group F			
SOX7	3.020±1.646	1.93	1.00
SOX17	0.690±0.362	1.37	0.97
SOX18	0.483±0.110	3.24	1.44

*This information was referenced from NCBI (https://www.ncbi.nlm.nih.gov/gene/).

**This information was referenced from RefEx (https://refex.dbcls.jp/).

Figure S1. Apolipoprotein genes form one topologically associating domain on human chromosome 11.

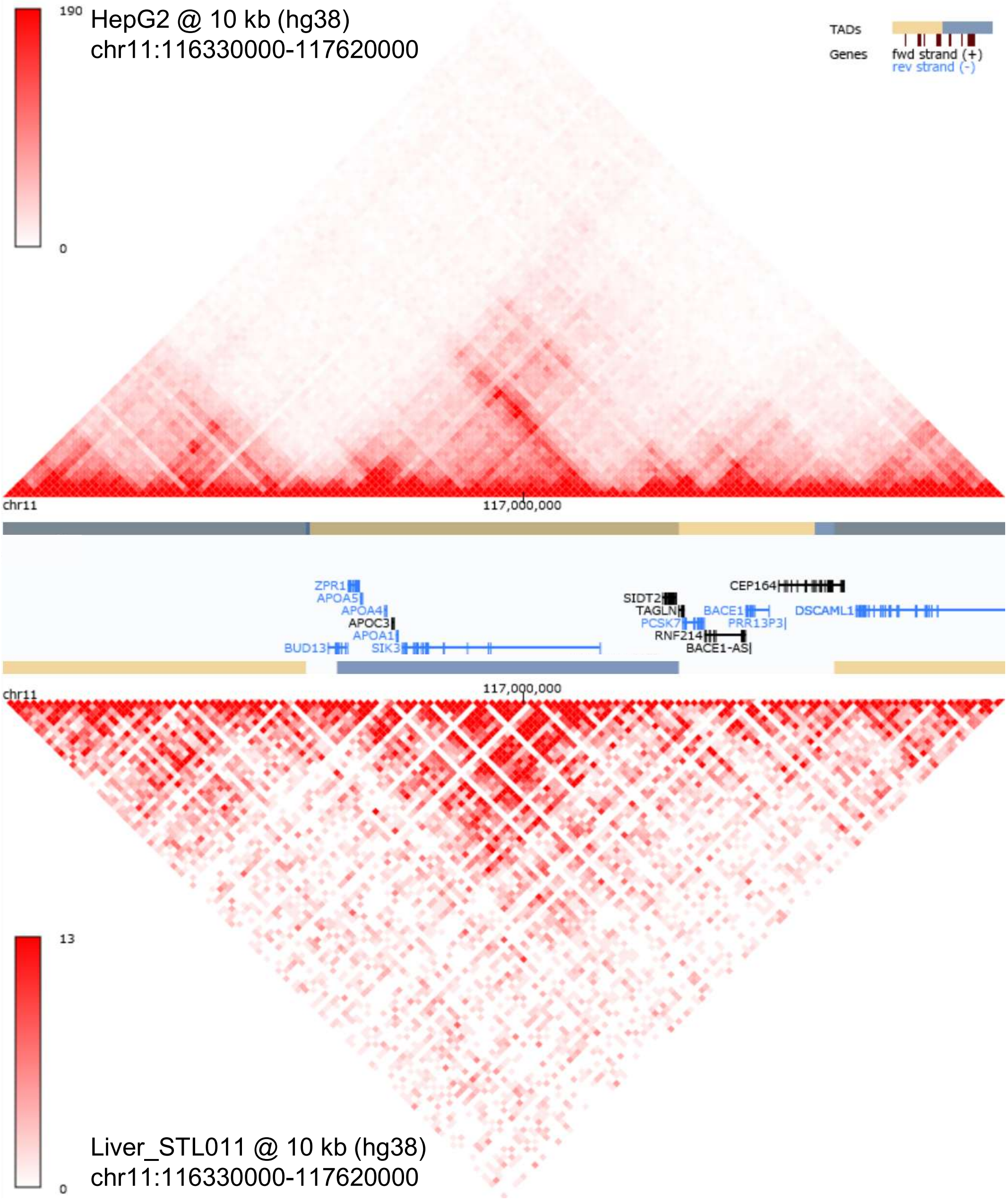

The figure was created on the YUE Lab website (https://3dgenome.fsm.northwestern.edu/compare.php) using two Hi-C data sets: HepG2 and Liver_STL011.

Figure S2. Endogenous APOA1 gene expression in HepG2, Hep3B, and HEK293 cells.

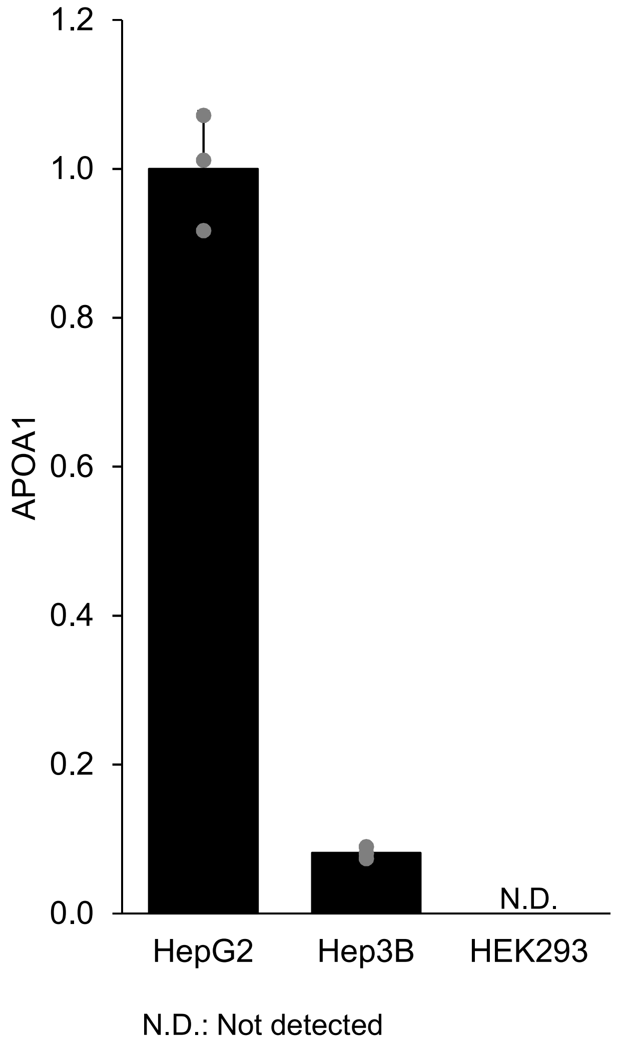

RNA was extracted from cells cultured under standard conditions, and endogenous APOA1 expression was quantified by reverse-transcription quantitative PCR.

Figure S3. Reporter-based screen in AML12, a mouse hepatocyte cell line.

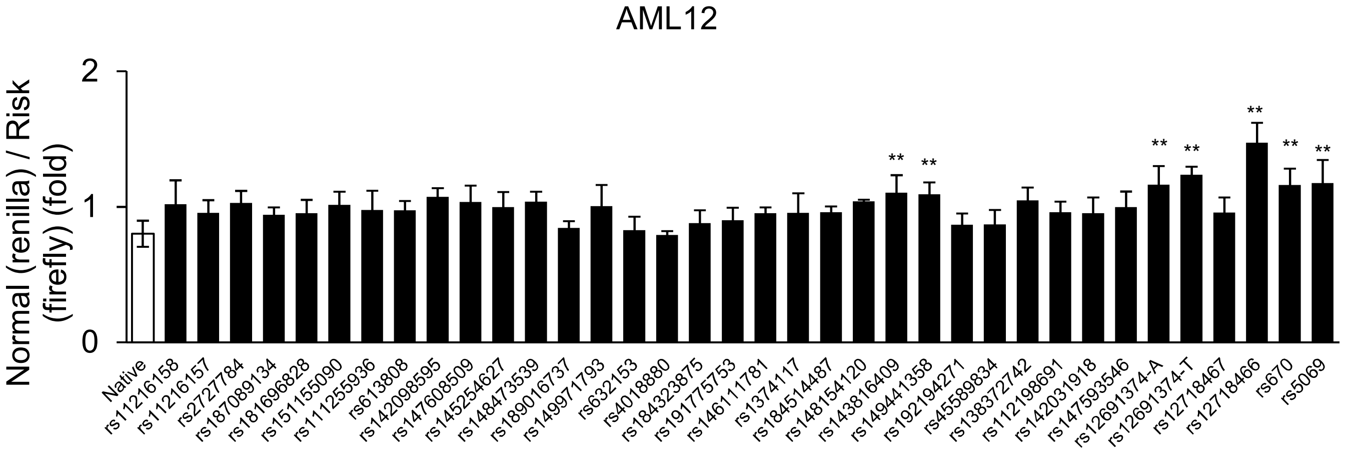

The reporter plasmids were co-transfected into AML12 cells, and luciferase activity was subsequently measured (n = 4). **: P < 0.01.

Figure S4. shRNA knockdown efficiency against hSox7 and mSox7.

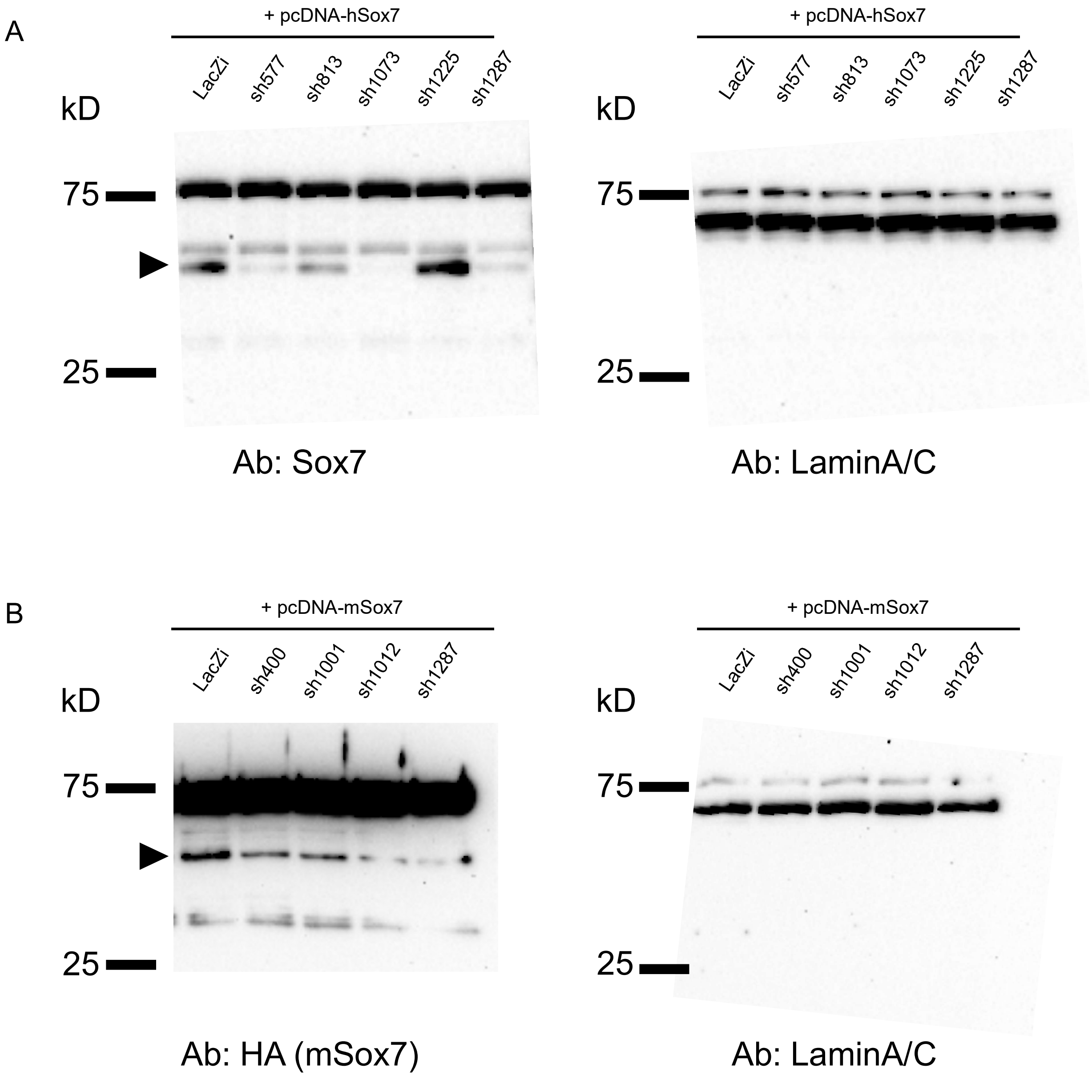

(A) shRNA knockdown efficiency against hSox7. We verified the knockdown effects of sh577 and sh1287 in vitro, and both were effective when delivered via adenovirus. In contrast, sh1073 showed no effect when delivered via adenovirus. (B) shRNA knockdown efficiency against mSox7. Western blotting was performed using the following primary antibodies: anti-SOX7 (sc-20093), anti-Lamin A/C (sc-376248), and anti-HA tag (sc-57592), all obtained from Santa Cruz Biotechnology. Secondary antibodies appropriate for each primary antibody were used.
